# Indonesian Translation and Cross-Cultural Validation of Pediatric Anesthesia Parent Satisfaction (PAPS) Questionnaire

**DOI:** 10.7759/cureus.36053

**Published:** 2023-03-12

**Authors:** Djayanti Sari, Yunita Widyastuti, Anisa Fadhila Farid, Mochamad Aldi Dwiyana, Anita Amalia

**Affiliations:** 1 Department of Anesthesiology and Therapy Intensive, Faculty of Medicine, Public Health, and Nursing, Universitas Gadjah Mada/Dr. Sardjito General Hospital, Yogyakarta, IDN; 2 College of Medicine, Faculty of Medicine, Public Health, and Nursing, Universitas Gadjah Mada, Yogyakarta, IDN

**Keywords:** parent’s satisfaction, reliability test, validity test, translation process, indonesian version, paps questionnaire

## Abstract

Background: Many questionnaires have been widely used to assess patient satisfaction. One of them is the Pediatric Anesthesia Parent Satisfaction (PAPS) questionnaire. However, in Indonesia, the parent satisfaction of pediatric patients undergoing surgery has never been studied. Knowing patient satisfaction can provide feedback to improve the quality of anesthesia services. Furthermore, the PAPS questionnaire has never been used in Indonesia, so it needs to be translated and tested for validity and reliability before being implemented in Indonesia.

Aim: to validate the Indonesian version of the PAPS questionnaire

Method: This study uses a descriptive-analytic method with a cross-sectional design. The PAPS Questionnaire was translated into Indonesian version with the following steps (1) forward translation, (2) establish an expert committee, and (3) backward translation. After that, pilot testing and FGD with the expert were conducted to obtain the final questionnaire. Pearson’s correlation and confirmatory factor analysis (CFA) were employed for the validity test. Sampling measurement before CFA was carried out with Keyser-Meyer-Olkin (KMO) and Bartlett’s test. Cronbach's alpha determined the reliability test evaluation.

Result: Total number of subjects is 125 subjects. The PAPS questionnaire in the Indonesian version was obtained with help from INCULS and an anesthesiologist expert. Pearson's correlation found that all items ranged from *r* = 0.531-0.796 with *p* < 0.001. The CFA showed two factors that explained 65% of the total variance, with KMO being 0.937 (*X2*=1283.452, *p *< 0.001). Cronbach’s alpha coefficient was 0.937.

Conclusion: Indonesian version of the PAPS questionnaire is valid and reliable for assessing parents' satisfaction with the anesthesia services their child received.

## Introduction

Anesthesia is an action to eliminate consciousness temporarily and makes the patient unable to feel sensations while maintaining the function of the autonomic nervous system, cardiovascular, respiratory, and thermoregulatory systems [1,2]. In addition to maintaining patient safety, administering anesthesia can also affect body conditions that contribute to morbidity and mortality [3]. Patients expect to receive good-quality anesthesia to feel satisfied with the services [4].

Patient satisfaction can be defined as the level of achievement the patient expects [5]. Satisfaction with anesthesia services is one indicator of anesthesia service quality [4]. An assessment of patient satisfaction needs to be undertaken by all facilities related to health facilities at this time. As health professionals have shifted to being "patient-centered," satisfaction has become essential to a quality assessment [6].

Many questionnaires have been widely used to assess patient satisfaction [7]. Some tools to evaluate the satisfaction levels of pediatric patients are the Child ZAP questionnaire, the Quality of Care Parent Questionnaire, A Modified Wong-Baker FACES Pain Rating Scale, and Pediatric Anesthesia Parent Satisfaction (PAPS) [8-10]. The PAPS is one of the most widely used questionnaires in many countries. Countries that use PAPS are Turkey and Ethiopia [11,12].

In Indonesia, parents’ satisfaction with anesthesia services for pediatric patients undergoing surgery has never been studied. Knowing the patient's satisfaction levels can provide feedback to improve the quality of anesthesia services. Therefore, it is necessary to evaluate the anesthesia services provided. Furthermore, the PAPS questionnaire has never been used in Indonesia, so it needs to be translated and tested for validity and reliability before applying in Indonesia. Due to differences in the language and culture in Indonesia, translation protocol is critical before entering the next test [13].

Due to the importance of measuring parent satisfaction with anesthesia services and the tools to assess it is still limited in Indonesia. Consequently, this study aims to establish the validity and reliability of the PAPS questionnaire in the Indonesian version.

## Materials and methods

Methodology

**Figure 1:**
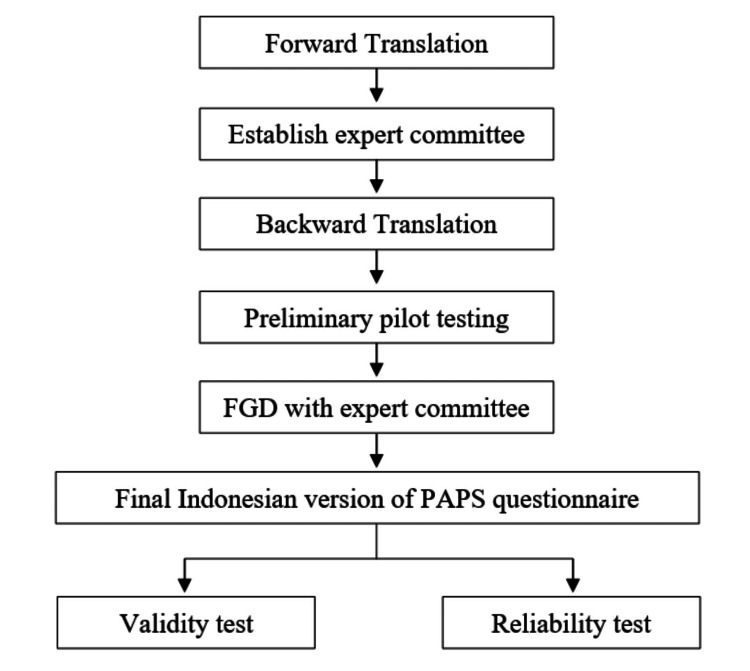
Step for translating questionnaire. FGD: Focused group discussions; PAPS: Pediatric anesthesia parent satisfaction

This study uses a descriptive-analytic method with a cross-sectional design. The study was conducted at Sardjito General Hospital. First, we followed some steps according to Tsang et al. for translating the PAPS Questionnaire from English to Indonesian version [13]. Figure 1 shows the steps we conducted in our study. This study was approved by the Medical and Health Research Ethics Committee (MHREC), Faculty of Medicine, Public Health, and Nursing, Universitas Gadjah Mada - Sardjito General Hospital, with approval number KE/FK/0818/EC/2020.

Participants

This study used consecutive sampling in which all the pediatric patient's parents who meet the inclusion and do not meet the exclusion criteria become the subject. The inclusion criteria in this study were (1) Parents of patients aged <18 years old who have undergone surgery under general anesthesia at Sardjito General Hospital, (2) ability to read and understand the Indonesian language, and (3) willingness to be a respondent. The exclusion criteria were (1) the patient's parent had difficulty communicating, and (2) the patient's parent had a pathological condition that made him/her unable to fill or complete the questionnaire.

According to Anthoine et al., there is no gold standard in determining the sample size of the validation study. However, most validation studies use a sample of three times the number of questions [14].

n = 3 x number of question = 3 x 15 = 45

n = minimum number of samples

Therefore, this study used a minimum sample size of 45 subjects.

PAPS questionnaire

The PAPS questionnaire consists of 17 questions with details of one question about the location of the operation, 15 closed questions, and one open question. The 15 open-ended questions will be assessed using a Likert scale with a scale of 1 to 5 (1 = strongly disagree, 2 = disagree, 3 = not sure, 4 = agree, 5 = strongly agree). At the end of the questionnaire, one open-ended question will contain the subject's general opinion of the anesthesia services provided [6].

Translation process

The PAPS Questionnaire was translated to the Indonesian version with help from The Indonesian Language and Culture Learning Services (INCULS) at the Faculty of Art and Humanities, Universitas Gadjah Mada. The first translation is entered readability test from the anesthesiology expert. Then, the questionnaire was translated back into English to match the meaning and content. Pilot testing was conducted to obtain input from respondents. In parallel, anesthesiologists hold Focused Group Discussions (FGD) to get feedback on the question that can be proposed to be added, changed, or reduced according to the conditions in Indonesia. The final PAPS questionnaire in Indonesia from FGD was used for data collection, and the validity and reliability test began.

Validity and reliability test

The validation test was computed using Pearson's correlation coefficient (r) [15]. The criteria for interpreting are as follows: strongly valid (r >0.35), likely to be useful (r = 0.21-0.35), depends on circumstances (r = 0.11-0.20), and unlikely to be useful (r < 0.11). Construct validity is the most critical concept in evaluating a questionnaire design. It measures the not directly observable construct (e.g., pain, quality of recovery) [13]. We evaluated the construct validity with Confirmatory Factor Analysis (CFA). Good constructs achieve when factor loading >0.40. Before CFA was conducted, Keyser-Meyer-Olkin (KMO) and Bartlett's test were conducted to measure sampling adequacy [16]. The KMO value was divided into several ranges: KMO ≥ 0.6 means low adequacy, KMO ≥ 0.7 means moderate adequacy, KMO ≥ 0.8 means high adequacy, and KMO ≥ 0.9 means very high [17]. The convergent validity of the items was determined using communalities. Each item was considered unrelated when a value <0.40, 0.40-0.70 was considered low to moderate communality, and a value >0.80 indicated high communality. When it has a value <0.40, the item must be excluded from the questionnaire [18].

Internal consistency reflects the extent to which questionnaire items are correlated with each other or are consistent in measuring the same construct. It is usually estimated using the alpha coefficient, known as Cronbach's Alpha. The criteria for interpreting an internal consistency reliability coefficient are as follows: excellent (≥ 0.90), good (0.80 - 0.90), adequate (0.70 - 0.79), and less applicable (< 70) [15].

The questionnaire is valid if Pearson’s correlation shows p <0.05 and has good construct validity (CFA >0.4) [15,16]. It was said to be reliable if Cronbach’s Alpha had a value of >0.70 [18]. In addition, the communality showed that each item had a relation to the other and was expected to have a value >0.40 [19].

Statistical analysis

We used IBM Corp. Released 2015. IBM SPSS Statistics for Windows, Version 23.0. Armonk, NY: IBM Corp. to analyze statistics in this study. Validity test using Pearson correlation test, and construct validities using CFA. Before CFA was conducted, to evaluate the sample adequacy, we used the KMO Measure of Sampling and Bartlett's test of sphericity. Internal consistency reliability using Cronbach's alpha.

## Results

Subject characteristics

The total number of subjects that met the inclusion and did not meet the exclusion criteria was 125 subjects. The patient and parent characteristics that we collected were age, gender, parent's education, parent occupation, ASA score, comorbidity, and history of operation. The patient and parent's characteristics are shown in Table 1.

**Table 1:** Pediatric Patients and Parent’s Characteristics. ASA: American Society of Anesthesiologists; n: Number; SD: Standard deviation

Parameter	Total
Pediatric Patients’ Characteristic	
Patient’s age (years), mean±SD	6.78±5.05
Patient’s Gender, n (%)	
Male, n (%)	70 (56.0)
Female, n (%)	55 (44.0)
Priority of Surgery, n (%)	
Elective, n (%)	121 (96.8)
Emergency, n (%)	4 (3.2)
ASA, n (%)	
I, n (%)	45 (36.0)
II, n (%)	71 (56.8)
III, n (%)	8 (6.4)
IV, n (%)	1 (0.8)
Comorbid, n (%)	
No, n (%)	106 (84.8)
Yes, n (%)	19 (15.2)
History of Operation, n (%)	
Never, n (%)	79 (63.2)
Ever, n (%)	46 (36.8)
Parents’ Characteristic	
Parent’s age (years), mean±SD	36.7±7.9
Patient’s Gender, n (%)	
Male, n (%)	62 (49.6)
Female, n (%)	63 (50.4)
Parent’s education, n (%)	
Elementary School, n (%)	13 (10.4)
Junior High School, n (%)	22 (17.6)
High School, n (%)	64 (51.2)
Bachelor’s degree, n (%)	24 (19.2)
Master’s degree, n (%)	2 (1.6)
Parent’s occupation, n (%)	
Unemployed, n (%)	33 (26.4)
Farmer/ merchant/ laborer, n (%)	19 (15.2)
Private employee/ self-employed/ shop owner, n (%)	50 (40.0)
Civil servant, n (%)	9 (7.2)
Other, n (%)	14 (11.2)

Translation process

Forward translation to the Indonesian version was carried out with the help of INCULS. An anesthesiology expert reads the first translation, and after getting some input, translation back to English is carried out to match the meaning and content by INCULS. Pilot testing was helped by four anesthesia consultants, five patients' families, six nurses, and seven anesthesia residents. The suggestions from this pilot testing are discussed in the FGD set by the expert.

Validity and reliability test

Content validity is conducted in FGD set by the expert, and the output is the final PAPS questionnaire in the Indonesian version. This version was assessed for validity and reliability. See Table 2.

**Table 2:** The Indonesian version of The Pediatric Anesthesia Parents Satisfaction (PAPS) Questionnaire.

Item	Original Version	Indonesian Version
1	The anesthesia team answered all my questions before my child's surgery.	Tim anestesi menjawab semua pertanyaan saya sebelum melakukan pembiusan kepada anak saya
2	I was satisfied with the amount of information provided by the anesthesia team.	Saya merasa puas atas semua informasi yang telah diberikan oleh tim anestesi
3	The information given to me by the anesthesia providers was understandable.	Informasi yang diberikan oleh tim anestesi dapat saya pahami dengan mudah
4	I was satisfied with the way my child fell asleep and woke up from anesthesia.	Saya merasa puas dengan cara anak saya tertidur dan sadar dari Pembiusan
5	The anesthesia team explained to me how my child might feel physically and emotionally after anesthesia and surgery.	Tim anestesi menjelaskan hal-hal yang mungkin dirasakan oleh anak saya baik secara fisik maupun emosional setelah dilakukan pembiusan dan operasi
6	I felt my child's pain was well controlled after surgery.	Saya merasa bahwa rasa sakit anak saya tertangani dengan baik setelah operasi
7	I felt my child's nausea and vomiting were well controlled after surgery.	Saya merasa jika anak saya mual dan muntah setelah operasi, anak saya tertangani dengan baik
8	My child's privacy was respected at all times by the anesthesia team.	Tim anestesi selalu menghormati privasi anak saya
9	The staff we met for my child's surgery behaved professionally and respectfully.	Tim anestesi yang kami temui untuk pembiusan anak saya bertindak secara profesional dan sopan
10	The anesthesia team behaved professionally and respectfully toward my child.	Tim anestesi bertindak secara profesional dan sopan terhadap anak saya
11	The anesthesia team paid attention to my concerns regarding my child's care.	Tim anestesi memperhatikan kekhawatiran saya terkait perawatan anak saya
12	I was satisfied with the care my child received from the anesthesia team.	Saya merasa puas atas perawatan yang diberikan oleh Tim Anestesi terhadap anak saya
13	I know who the anesthesiologist (physician) was and his/her role in my child's care.	Saya tahu siapa dokter anestesi dan perannya dalam perawatan anak saya
14	I would recommend this anesthesia team to others in my family.	Saya akan merekomendasikan tim anestesi ini kepada anggota keluarga saya
15	My child received the highest quality care during this surgical experience.	Anak saya mendapat perawatan terbaik selama proses pembiusan

The results of our validity study showed that the Indonesian version had a strong validity (r > 0.35) and p <0.001, see Table 3.

**Table 3:** Indonesian version validity test for each item of the questionnaire. *p<0.05

Component	r	p-value	Interpretation (r > 0.35)
Item 1	0.777	<0.001*	Strongly valid
Item 2	0.739	<0.001*	Strongly valid
Item 3	0.752	<0.001*	Strongly valid
Item 4	0.725	<0.001*	Strongly valid
Item 5	0.791	<0.001*	Strongly valid
Item 6	0.768	<0.001*	Strongly valid
Item 7	0.686	<0.001*	Strongly valid
Item 8	0.796	<0.001*	Strongly valid
Item 9	0.765	<0.001*	Strongly valid
Item 10	0.785	<0.001*	Strongly valid
Item 11	0.796	<0.001*	Strongly valid
Item 12	0.794	<0.001*	Strongly valid
Item 13	0.785	<0.001*	Strongly valid
Item 14	0.531	<0.001*	Strongly valid
Item 15	0.710	<0.001*	Strongly valid

The KMO of 0.937 (X2 = 1283.452, p < 0.001) was very high sampling adequacy; therefore, the sample was suitable to be analyzed by CFA. The CFA showed compatibility with the existence of two factors that explained 64.985% or 0.65 of the total variance; it indicated a good construct (factor loading >0.40), see Table 4. The communality value of 15 items was 0.449-0.797, indicating that the relationship with other items had low to moderate communality, see Table 5.

**Table 4:** Total variance explained by the Confirmatory Factor Analysis. Extraction sums of square loadings are obtained from all the factor loadings with total initial eigenvalues >1.000, so the factors loadings that meet the criteria are item 1 and item 2 with the total variances 56.612% + 8.373% = 64.985% or 0.65.

Component	Initial Eigenvalues			Extraction Sums of Squared Loadings		
	Total	% of variance	Cumulative %	Total	% of variance	Cumulative %
Item 1	8.492	56.612	56.612	8.492	56.612	56.612
Item 2	1.256	8.373	64.985	1.256	8.373	64.985
Item 3	0.880	5.865	70.850			
Item 4	0.658	4.387	75.237			
Item 5	0.522	3.483	78.720			
Item 6	0.480	3.203	81.923			
Item 7	0.427	2.845	84.768			
Item 8	0.406	2.703	87.471			
Item 9	0.399	2.661	90.132			
Item 10	0.329	2.191	92.323			
Item 11	0.317	2.114	94.437			
Item 12	0.280	1.865	96.302			
Item 13	0.221	1.475	97.777			
Item 14	0.170	1.133	98.910			
Item 15	0.164	1.090	100.000			

**Table 5:** Communalities values for all items.

Component	Initial	Extraction
Item 1	1.000	0.615
Item 2	1.000	0.645
Item 3	1.000	0.616
Item 4	1.000	0.608
Item 5	1.000	0.624
Item 6	1.000	0.588
Item 7	1.000	0.449
Item 8	1.000	0.745
Item 9	1.000	0.755
Item 10	1.000	0.746
Item 11	1.000	0.669
Item 12	1.000	0.642
Item 13	1.000	0.690
Item 14	1.000	0.797
Item 15	1.000	0.558

A reliability test was carried out using Cronbach’s Alpha. Based on the analysis results, Cronbach's alpha value of 15 items was 0.937. This indicates that the questionnaire was reliable with excellent internal consistency.

## Discussion

Patient satisfaction is an essential aspect of assessing the quality of health services [20]. In pediatric patients, knowing satisfaction with the services provided is difficult. This is because pediatric patients, especially at a younger age, have not been able to express how they feel, so parents represent the satisfaction assessment through the PAPS questionnaire [6,21]. In Indonesia, an assessment of the satisfaction level of anesthesia services has been carried out by the multidimensional Leiden Perioperative Patient Satisfaction Questionnaire (LPPSq) to assess general and regional anesthesia services [22,23]. The use of the PAPS questionnaire for parents of pediatric patients undergoing anesthesia has never been done in Indonesia [6,11,12]. So, in this study, it is necessary to translate and test it for validity and reliability before assessing the level of satisfaction with anesthesia services for children. Translation into the Indonesian version is needed because the original PAPS questionnaires were in English, and the research took place in America, a developed country [6]. Their culture differs from other countries, especially in Indonesia [24]. This provides a more solid basis for why translation in different cultures is necessary.

In our study, the PAPS questionnaire in the Indonesian version is valid and reliable for measuring parent satisfaction when their child undergoes surgery, especially by measuring anesthesia services. The Indonesian version of the PAPS questionnaire has strong validity, good construct validity, low-to-moderate communality, and excellent internal consistency. These results align with the original study for the PAPS questionnaire conducted by Miliken-Galbe in 2017 [6]. In this study, the validity and reliability values were obtained with Cronbach's alpha and Raykov's rho, in which the value was > 0.7. Our research differs from previous studies, namely in the understanding and meaning of the word "Parents." In previous studies, the word "Parent" referred to the biological parents of the patient, while in this study, the word "Parent" has a broader meaning and can include caregivers, guardians, or the patient's closest person who accompanied the patient before the surgery until the surgery is done [6].

The PAPS questionnaire is one of the questionnaires related to parent satisfaction. It has several advantages over other questionnaires, including its simplicity to fill out and its high level of readability, which makes it easy for respondents with a low level of education to comprehend. In addition, the questions in the PAPS questionnaire are a direct adaptation of a series of questions recommended by the American Society of Anesthesiologists Committee on Performance and Outcomes Measurement (ASA COPM) and cover several essential aspects such as symptoms, communication, and professionalism [6]. By knowing the level of satisfaction of the patient's parents, the hospital or other healthcare providers can find out which aspects require quality improvement, especially for pediatric patients. In addition, the PAPS questionnaire can be used to know parent satisfaction, even the patients having a developmental delay [11].

Two countries study the validation of the PAPS questionnaire, Turkey and Ethiopia. In Ethiopia, they investigated the correlation between parental background and the parent's satisfaction with the anesthesia services their child received [12]. In addition, in Turkey, they assess the parent’s satisfaction with anesthesia care in pediatric patients with and without developmental delay. Those two countries have successfully implemented the PAPS questionnaire in their own countries. Our study may be one of a reference to see that the PAPS questionnaire can be used in other languages and even cross-cultural.

As explained above, Indonesia and America have different cultures. In developed countries, such as America, parents are more tend to anxious when their children are being anesthetized than in developing countries, such as Indonesia [24]. This difference is due to the differences in parenting styles in Western and Indonesian cultures. In western cultures, they tend to be more authoritative (high demand and high responsive). However, in Indonesia, parents are more authoritarian (high demand and less responsive). This causes differences in the mindset of parents and children [25]. In addition, the differences in culture are a determinant factor of parent satisfaction, and validation of questionnaires with differences in culture needs to be carried out, and this requires proper guidelines for the translation and validation process [13,24].

Our study has several limitations. First, we used a small sample size that may not apply to the large population. Further research conducted with a larger sample size is needed. In addition, the concurrent validity, which is part of the validity and reliability test, was not carried out because it was outside the aim and scope of our study. Further research on the concurrent validity of the PAPS questionnaire is needed.

## Conclusions

The Indonesian version of the PAPS questionnaire is valid and reliable in assessing the parent’s satisfaction with the anesthesia services their child receives. It has strong validity, good construct, low to moderate communality, and excellent internal consistency. In addition, it is mandatory to use appropriate guidelines for translating and validating the questionnaire in other languages ​​and cultures. Further research on the concurrent validity of the PAPS questionnaire is recommended.
